# Pathologic, Laboratory, and Surgical Findings of Topical Statin Gels (Simvastatin, Atorvastatin, and Rosuvastatin) in a Rat Model of Peritoneal Endometriosis

**DOI:** 10.3390/gels12030201

**Published:** 2026-02-28

**Authors:** Shahla Chaichian, Roya Derakhshan, Samaneh Rokhgireh, Amirhossein Larijani, Arash Bakhshi, Abolfazl Mehdizadehkashi, Marziyeh Ajdary, Mohammad Abbas Sheikholeslami, Behrang Kazeminezhad, Seyed Ali Ziai, Babak Sabet

**Affiliations:** 1 Endometriosis Research Center, Iran University of Medical Sciences, Tehran 1449614535, Iran; shchaichian@gmail.com (S.C.); s.rokhgireh@gmail.com (S.R.); amehdizadehkashi@yahoo.com (A.M.); maa.biology92@gmail.com (M.A.); 2 Pars Advanced and Minimally Invasive Medical Manners Research Center, Pars Hospital, Iran University of Medical Sciences, Tehran 1449614535, Iran; 3 Student Research Committee, Guilan University of Medical Sciences, Rasht 41625, Iran; amir1313801360@gmail.com (A.L.); a.bakhshi.b13@gmail.com (A.B.); 4 Department of Pharmacology, School of Medicine, Shahid Beheshti University of Medical Sciences, Tehran 1983535511, Iran; masheikh@sbmu.ac.ir; 5 Department of Pathology, Clinical Research Development Center, Shahid Modarres Educational Hospital, Shahid Beheshti University of Medical Sciences, Tehran 1983535511, Iran; dkazeminezhad@gmail.com; 6 Department of Pharmacology, Faculty of Medicine, Shahid Beheshti University of Medical Sciences, Tehran 1983535511, Iran; aliziai@sbmu.ac.ir; 7 Department of Surgery, Faculty of Medicine, Shahid Beheshti University of Medical Sciences, Tehran 1983535511, Iran; 8 Artifical Intelligence in Medical Sciences Research Center, Smart University of Medical Sciences, Tehran 1587616415, Iran

**Keywords:** topical statin gels, simvastatin, atorvastatin, rosuvastatin, peritoneal endometriosis, rat model, postoperative adhesion, anti-inflammatory effect

## Abstract

Endometriosis is a chronic inflammatory disease with frequent recurrence. Statins, due to their anti-inflammatory and antioxidant properties, may help control disease progression, but comparative data on local administration are limited. We evaluated simvastatin-, atorvastatin-, and rosuvastatin-loaded lipophilic gels on lesions, adhesions, and inflammatory markers in a rat model of peritoneal endometriosis. Forty rats were randomized to statin gels (*n* = 10 each), chitosan gel (vehicle; *n* = 5), or no treatment (control; *n* = 5). Two weeks later, lesion size, adhesions, histopathology, and serum Interleukin-6 (IL-6) and Interleukin-1β (IL-1β) were assessed. All statins significantly reduced endometriotic lesion size compared with controls. Lesion volume decreased by approximately 97% with simvastatin, 88% with atorvastatin, and 72% with rosuvastatin, whereas lesion volume increased in the control and vehicle-treated groups. Adhesion severity was markedly reduced, with Hoffman scores decreasing from 7.2 ± 2.25 in controls to 1.9 ± 1.1 with simvastatin, compared with more modest reductions observed with atorvastatin and rosuvastatin. Similarly, Lauder adhesion scores were reduced by approximately 71% with simvastatin, confirming its superior anti-adhesion effect. Serum IL-6 and IL-1β levels were significantly decreased in all statin-treated groups, with no significant differences among statins. Overall, topical statin gel therapy effectively reduced lesion size, adhesions, and inflammation, with simvastatin showing superior anti-adhesion effects.

## 1. Introduction

Endometriosis affects approximately 6–10% of women of reproductive age. It presents with a wide range of symptoms, including dysmenorrhea, dyspareunia, absence of menstrual pelvic pain, dyschezia, gastrointestinal disturbances, urinary or renal symptoms, and infertility. This condition significantly impacts women’s daily functioning, occupational performance, and overall well-being, thereby reducing quality of life [1]. Women with endometriosis often require long-term treatment due to the chronic and recurrent nature of the disease. Despite available medical and surgical options, current therapies are not curative, and symptom recurrence is common even after initial treatment success. Previous studies have shown that many patients are willing to tolerate adverse effects of medical therapy in exchange for effective pain relief, highlighting the unmet need for safer and more effective therapeutic strategies [2,3].

Additionally, it has been shown that patients with endometriosis often require a second or even third surgical intervention during follow-up, reflecting the chronic and recurrent nature of the disease [4].

Oxidative stress is believed to play a crucial role in the initiation and progression of endometriotic lesions. Excessive production of reactive oxygen species (ROS), generated during normal oxygen metabolism, has been implicated in the pathophysiology of endometriosis by promoting the growth, adhesion, and survival of endometrial cells within the peritoneal cavity. ROS induce cellular damage through lipid peroxidation, increased membrane permeability, and DNA injury, thereby contributing to tissue remodeling and disease progression. Consequently, antioxidant and anti-inflammatory agents have attracted increasing interest as potential therapeutic options [5,6,7]. Statins, widely used lipid-lowering agents, exhibit pleiotropic effects, including antioxidant, anti-inflammatory, antiproliferative, and anti-angiogenic properties, making them attractive candidates for endometriosis management [8]. Experimental studies have demonstrated that statins can inhibit endometrial cell proliferation, suppress angiogenesis, and modulate fibrinolytic activity in both in vitro and animal models of endometriosis. These effects have been associated with reductions in lesion size and attenuation of postoperative adhesion formation, although outcomes appear to vary depending on statin type, dosage, and route of administration. However, most available studies have evaluated individual statins in isolation or used systemic administration approaches, limiting direct comparison of their relative efficacy [9,10,11].

Additionally, simvastatin potently enhances local peritoneal fibrinolytic activity by upregulating tissue plasminogen activator (t-PA) levels and downregulating plasminogen activator inhibitor-1 (PAI-1) expression in mesothelial cells [12]. Despite accumulating evidence supporting the therapeutic potential of statins in endometriosis, a critical knowledge gap remains regarding the comparative effectiveness of commonly used statins when administered locally under standardized experimental conditions [13]. Moreover, the potential role of gel-based local statin delivery—particularly in the context of surgical management of endometriosis—has received limited attention.

Based on these considerations, the present study was conducted to compare the therapeutic efficacy of simvastatin, atorvastatin, and rosuvastatin in the treatment of peritoneal endometriosis using a single, standardized experimental model with local gel-based administration [14].

We hypothesized that local delivery of statins would differentially reduce endometriotic lesion burden and postoperative adhesion formation, with variations reflecting differences in their pharmacologic and pleiotropic profiles.

## 2. Results and Discussion

### 2.1. Effects of the Studied Drugs on the Size and Volume of Endometriotic Tissue

**Figure 1:**
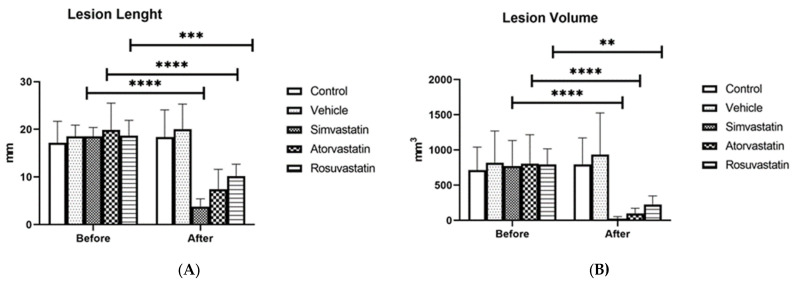
Effects of simvastatin, atorvastatin, rosuvastatin, and vehicle on the length and volume of endometriotic lesions in experimental groups: (**A**) comparison of lesion length; (**B**) comparison of lesion volume. Before: before treatment, After: after treatment. ** *p* < 0.01, *** *p* < 0.001, **** *p* < 0.0001.

As illustrated in Figure 1, all tested statins reduced both the length (Figure 1A) and volume (Figure 1B) of peritoneal endometriotic lesions compared with the vehicle (chitosan) and control group. The reduction in lesion length was statistically significant in all treatment groups (*p* = 0.0001), with no significant difference among simvastatin, atorvastatin, and rosuvastatin.

Regarding lesion volume, all three drugs significantly decreased the size of endometriotic implants; however, the effect of rosuvastatin (*p* = 0.0005) was slightly less pronounced than that of simvastatin and atorvastatin (*p* = 0.0001) (Table 1).

### 2.2. Effects of the Studied Drugs on Adhesion Formation

As shown in Table 2, all statins significantly reduced adhesion formation according to the Lauder scoring criteria when compared with the control group, with simvastatin demonstrating the most substantial reduction (*p* < 0.0001). Based on the Hoffmann scoring criteria, only simvastatin showed a statistically significant decrease in adhesion severity relative to the control group (*p* < 0.002). The chitosan-treated (vehicle) group exhibited no significant difference from untreated controls.

**Figure 2:**
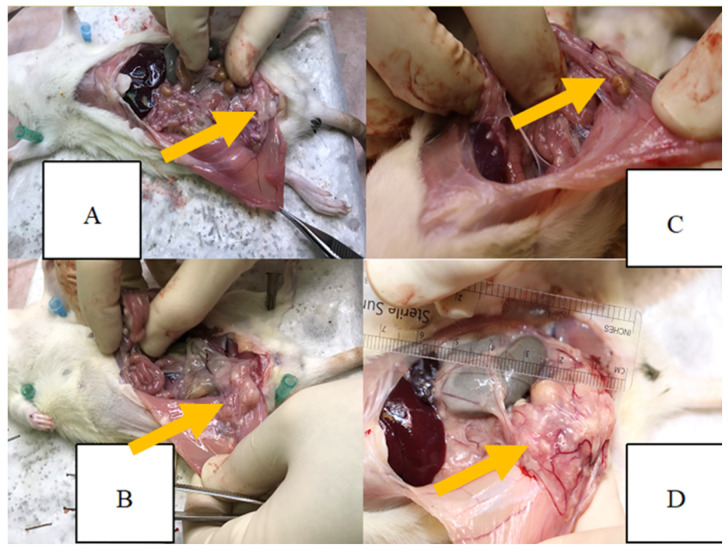
Severity of adhesions in the four experimental groups at the end of the study: (**A**) simvastatin-treated group; (**B**) atorvastatin-treated group; (**C**) rosuvastatin-treated group; (**D**) chitosan-treated control group. Yellow arrows show the endometriotic lesion.

At the end of the study, the size of endometriotic lesions and the severity of adhesions differed among the experimental groups. As shown in Figure 2, rats treated with simvastatin, atorvastatin, and rosuvastatin gels exhibited reduced adhesion severity compared with the chitosan-treated group.

**Figure 3:**
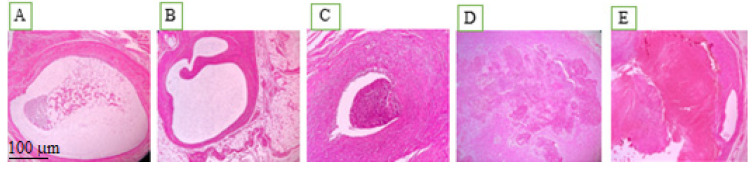
Pathological views of ectopic endometriosis in the experimental groups: (**A**) post-induction of endometriosis; (**B**) chitosan-treated control group; (**C**) rosuvastatin-treated group; (**D**) simvastatin-treated group; (**E**) atorvastatin-treated group.

Histopathologic analysis indicated variations in adhesion, inflammatory cell infiltration, and fibrosis between the study groups (Figure 3). Figure 3 depicts representative histologic characteristics found in each group, demonstrating variable degrees of adhesions, inflammation, and fibrotic alterations. Endometriosis was diagnosed histologically as the presence of ectopic endometrial glands and stroma with epithelial lining and luminal development [15].

### 2.3. Effects of the Studied Drugs on Inflammatory Cytokine Levels

Analysis of inflammatory markers demonstrated significant changes in cytokine levels between the intervention and control groups from the pre-induction phase to the end of the study. As shown in Table 3, serum levels of the inflammatory cytokines interleukin-6 (IL-6) and interleukin-1β (IL-1β) were significantly higher in the control group following surgery and induction of endometriosis compared with all treatment groups, as determined by the Kruskal–Wallis test. Two weeks after the initiation of statin therapy, levels of both cytokines were markedly reduced in all treated groups relative to the chitosan-treated and control groups; however, no significant differences were detected among the three statin-treated groups, indicating a substantial anti-inflammatory effect of the studied statins. Pairwise post hoc analysis revealed that all statin-treated groups differed significantly from the control groups; however, no statistically significant differences were observed among the statin treatment groups for inflammatory cytokine levels (all *p* > 0.05).

### 2.4. Discussion

In this study, we evaluated the effects of three lipophilic statins—simvastatin, atorvastatin, and rosuvastatin—delivered as topical gels on peritoneal endometriotic lesion size, postoperative adhesion formation, and inflammatory cytokine levels. All three statins significantly reduced lesion size and adhesion severity and promoted anti-inflammatory effects. That supports the hypothesis that statins may suppress ectopic endometrial growth, potentially through inhibition of inflammatory pathways, angiogenesis, and cellular proliferation.

Consistent with our observations, previous animal studies have shown that systemic statin administration reduces the growth of endometriotic implants by suppressing cellular proliferation, angiogenesis, and inflammatory mediators such as monocyte chemoattractant protein-1 (MCP-1) and matrix metalloproteinase-3 (MMP-3) in experimental models of endometriosis [16].

Additionally, statins have demonstrated anti-adhesion properties in peritoneal adhesion models, effects that are thought to occur through upregulation of local fibrinolytic activity and downregulation of pro-inflammatory pathways [17].

Further experimental evidence supports the dual anti-endometriotic and anti-adhesion effects of statins. In a nude mouse model, simvastatin treatment produced a dose-dependent decrease in both the number and size of endometriotic implants, with maximal inhibition of lesion growth at higher doses and associated reductions in matrix metalloproteinase 3 expression, a key mediator of extracellular matrix remodeling in endometriosis [11,14].

Similarly, in models of postoperative peritoneal adhesions, intraperitoneal administration of statins, including lovastatin and atorvastatin, significantly reduced adhesion formation, likely through enhanced fibrinolytic activity mediated by increased tissue plasminogen activator levels and modulation of plasminogen activator inhibitor-1 [18].

Additionally, intraperitoneal rosuvastatin has been reported to prevent postoperative peritoneal adhesion formation in rats, likely through inhibition of pro-inflammatory cytokine release such as TNF-α and IL-1α, a finding that parallels our observed reductions in adhesion severity and systemic IL-1β [19].

Based on our findings, although all three statins produced comparable reductions in lesion length, rosuvastatin demonstrated a slightly lesser effect on lesion volume compared with simvastatin and atorvastatin.

This difference may be explained by variations in statin lipophilicity and tissue penetration. Lipophilic statins such as simvastatin and atorvastatin can passively diffuse across cell membranes and achieve broader tissue distribution, whereas hydrophilic statins like rosuvastatin exhibit more limited extrahepatic uptake and rely on transporter-mediated cellular entry. These pharmacokinetic differences may contribute to the greater local therapeutic efficacy observed with lipophilic statins [20].

In addition to reducing lesion size, all statins significantly decreased postoperative adhesion formation, a clinically relevant complication of endometriosis surgery. Notably, simvastatin exhibited the most pronounced anti-adhesion effect, achieving significant reductions in adhesion severity according to both the Lauder and Hoffmann scoring systems. In contrast, atorvastatin and rosuvastatin reduced adhesion extent but did not significantly improve adhesion severity based on the Hoffmann criteria. These findings suggest that simvastatin may exert superior effects on peritoneal healing and fibrosis modulation.

Supporting these findings, a recent systematic review reported that statins consistently reduce both gross and microscopic adhesion scores in animal models, potentially through enhancement of fibrinolytic activity and modulation of extracellular matrix components such as MMP-9 [21]. Collectively, these data align with our results and highlight multiple complementary mechanisms, including anti-inflammatory, anti-proliferative, and pro-fibrinolytic pathways, through which locally delivered statin gels may exert therapeutic benefits in endometriosis and postoperative adhesion prevention.

From a clinical perspective, the prevention of postoperative adhesions remains a critical unmet need in gynecologic surgery, particularly in patients with endometriosis. Adhesion formation is strongly associated with chronic pelvic pain, dyspareunia, infertility, and impaired quality of life and can adversely affect reproductive outcomes in women desiring fertility [22]. Furthermore, postoperative adhesions may complicate future surgical procedures and increase the risk of bowel obstruction and surgical morbidity [23]. These clinical burdens underscore the importance of developing effective interventions to reduce adhesion formation and limit long-term disease-related sequelae [24].

The novel application of statins as topical intraperitoneal gel formulations may offer important advantages over systemic therapy by achieving higher local drug concentrations at pathological sites while minimizing systemic exposure.

Intraoperative use of statin-loaded gels could therefore represent a practical adjunctive strategy to reduce postoperative adhesion formation and potentially suppress residual endometriotic lesions following surgical excision. Nevertheless, further studies are required to optimize formulation characteristics, dosing, and delivery routes and to establish safety and efficacy in human clinical trials. The direct comparison of three statins under standardized experimental conditions in the present study further highlights differences in their relative effectiveness, with simvastatin demonstrating the strongest anti-adhesion effect [25].

## 3. Conclusions

Our findings suggest that the topical application of statin gel during surgery in patients with endometriosis may represent a promising adjunctive approach for reducing postoperative adhesion formation. In this study, topical statin gel significantly decreased adhesion scores at the end of the study and was associated with lower levels of inflammatory cytokines, indicating a parallel anti-inflammatory effect.

## 4. Materials and Methods

**Figure 4:**
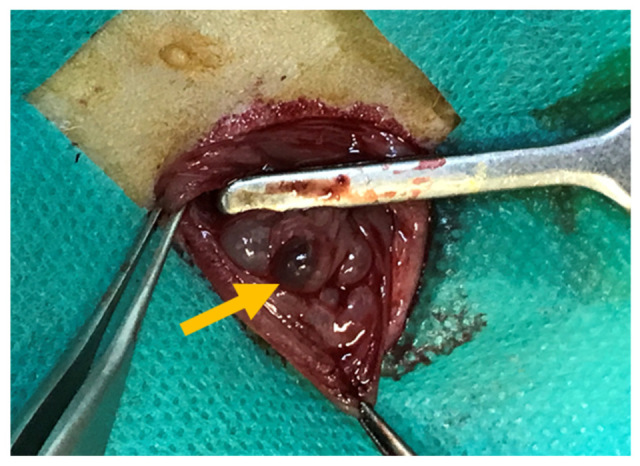
Induction of peritoneal endometriosis in experimental rats at baseline. The yellow arrow shows the endometriotic lesion.

This experimental study was conducted on 40 adult female rats of the same strain, aged 8 weeks and weighing 220–250 g. Peritoneal endometriosis was induced through autologous endometrial transplantation, as described below. Animals were anesthetized via intraperitoneal injection of ketamine (100 mg/kg; Exir, Tehran, Iran) and xylazine (10 mg/kg; Exir, Tehran, Iran). A 3 cm midline laparotomy was performed; the right uterine horn was ligated with 4-0 silk (SUPA, Tehran, Iran) suture, excised, and placed in phosphate-buffered saline (placed in phosphate-buffered saline (PBS; Sigma-Aldrich, St. Louis, MO, USA). The uterine horn was then opened longitudinally, divided into three sections, and each segment was sutured to the peritoneal surface of the left abdominal wall using nonabsorbable 4-0 silk (SUPA, Tehran, Iran) sutures. During the procedure, the operative field was kept moist with normal saline to prevent tissue desiccation. Before abdominal closure, 2 mL of normal saline was instilled into the peritoneal cavity, and the abdominal wall was closed in two layers using 2-0 polyglactin (SUPA, Tehran, Iran) sutures (Figure 4). All rats received two subcutaneous doses of estradiol, one preoperatively and one postoperatively.

Statin-loaded lipophilic gels were prepared using a chitosan–gelatin (Alfa Aesar, Ward Hill, MA, USA) matrix. 2 g of chitosan was dissolved in 50 mL of sterile 1% acetic acid acetic acid (glacial, 100%; Merck, Darmstadt, Germany) under constant stirring (400 rpm) at 37 °C. Ethylene glycol (1 mL; Neutron Pharmaceutical Co., Tehran, Iran) was then added, followed by the gradual incorporation of 3 g of gelatin until a homogeneous gel was obtained. Atorvastatin, rosuvastatin, or simvastatin (Sobhan Darou Co., Tehran, Iran) was subsequently incorporated to achieve a final concentration of 10% (*w*/*v*), and the mixture was stirred for an additional 2 h. All procedures were performed under sterile conditions.

After 21 days, a second laparotomy was performed for macroscopic assessment and biopsy of endometriotic lesions. Endometriotic lesions were located and quantified in three dimensions (length, width, and height in millimeters) with a caliper. The volume of each lesion was calculated using the prolate ellipsoid formula V (mm^3^) = 0.52 × width × length × height. Digital photographs of the lesions were taken, and all measurements were documented [15].

Forty rats were randomly allocated using a computer-generated random number list into five groups: Groups I–III (*n* = 10 each) received intraperitoneal administration of 1 mL of simvastatin-, atorvastatin-, or rosuvastatin-loaded gel, respectively; Group IV (*n* = 5) received 1 mL of chitosan gel (vehicle control); and Group V (*n* = 5) served as the untreated control and received no medication.

### 4.1. Assessment of Adhesion Formation

This observational study employed pathological findings of endometriosis and laboratory markers of inflammation (including serum interleukins IL-6 and IL-1β) measured using the enzyme-linked immunosorbent assay (ELISA) method. Two weeks after the initiation of drug therapy (i.e., five weeks after induction of endometriosis), a third laparotomy was performed. Adhesion formation was evaluated using the Hoffmann quantitative and Lauder qualitative scoring systems, supported by intraoperative photographs. Endometrial implants were excised, fixed in 10% formalin, sectioned at 4 µm, and stained with hematoxylin and eosin (H&E) for histopathologic examination. Findings were compared across all study groups. The surgeon performing the procedures, the biochemist, the histopathologist assessing the histology, and the statistician were all blinded to the group allocation. Adhesion scoring and histological outcome assessment were therefore conducted under blinded conditions.

### 4.2. ELISA Test

Inflammatory markers were measured using ELISA. For this purpose, serum concentrations of IL-6 and IL-1β were quantified using commercial ELISA kits (Karmania Pars Gen, Kerman, Iran). Blood samples were collected from the tail at three time points: (1) before surgery, (2) after induction of endometriosis, and (3) two weeks after treatment initiation. Samples were immediately placed on ice and stored at −80 °C until analysis. After collecting samples, serum was separated and analyzed according to the manufacturer’s instructions to determine cytokine concentrations. According to the manufacturer’s specifications, the sensitivity of the assays was 2 pg/mL for both IL-6 and IL-1β, with intra-assay and inter-assay coefficients of variation below 3% and 8%, respectively. Standard concentrations provided with the kits ranged from 2 to 122 pg/mL.

### 4.3. Statistical Analysis

All data were entered into IBM SPSS software, version 26 (IBM Corp., Armonk, NY, USA), for statistical analysis. Endometriotic lesion size was compared between groups using the Mann–Whitney U test, while adhesion severity was analyzed with the Kruskal–Wallis test. Additional analyses were performed in SPSS version 26 using independent *t*-tests and Kruskal–Wallis tests for ordinal variables.

## Figures and Tables

**Table 1:** Comparison of endometriotic lesion size and volume among the study groups before and after treatment.

	Lesion Measurements	Lesion Length (mm)		Lesion Volume (mm^3^)	
Study Groups		Before Treatment	After Treatment	Before Treatment	After Treatment
Simvastatin Mean ± SD		18.5 ± 1.9	3.8 ± 1.6	772.5 ± 362.3	24.7 ± 30.1
Atorvastatin Mean ± SD		19.9 ± 5.6	7.4 ± 4.2	804.1 ± 412.5	96.77 ± 75.18
Rosuvastatin Mean ± SD		18.7 ± 3.2	10.2 ± 2.5	795.2 ± 219.8	225 ± 122.16
Chitosan-treated Mean ± SD		18.5 ± 2.4	20 ± 5.3	819.5 ± 450.1	936.62 ± 591.68
Control Mean ± SD		17.2 ± 4.5	18.4 ± 5.7	713.96 ± 326.1	795.86 ± 375.3
*p* value		0.6	<0.0001	0.9	<0.0001

**Table 2:** Evaluation of adhesion severity in the different experimental groups according to the Lauder and Hoffmann scoring systems.

Groups	Simvastatin Mean ± SD	Atorvastatin Mean ± SD	Rosuvastatin Mean ± SD	Chitosan-Treated Mean ± SD	Control Mean ±SD	*p* Value
Hoffman Adhesion Score	1.9 ± 1.1	3.3 ± 1.4	3.5 ± 2.2	5.9 ± 1.5	7.2 ± 2.25	<0.0001
Lauder Adhesion Score	1 ± 0.4	2.7 ± 1.2	2.2 ± 1.5	2.4 ± 1.1	3.4 ± 1.5	<0.002

**Table 3:** Serum levels of IL-6 and IL-1β across experimental groups at different time points. Data are presented as mean ± SD. *p*-values for the after-treatment comparisons were calculated using the Kruskal–Wallis test. IL-6: interleukin-6; IL-1β: interleukin-1β.

*p*-Value (Post)	After Treatment	Before Treatment	Baseline	Group	Cytokine
<0.0001	1.6 ± 0.1	3.4 ± 0.5	2.7 ± 0.5	Simvastatin Mean ± SD	IL-6
	1.8 ± 0.3	3.3 ± 0.5	2.5 ± 0.4	Atorvastatin Mean ± SD	
	1.7 ± 0.03	3.2 ± 0.4	2.6 ± 0.7	Rosuvastatin Mean ± SD	
	2.9 ± 0.2	2.7 ± 0.4	2.9 ± 0.6	Chitosan-treated Mean ± SD	
	2.7 ± 0.4	2.7 ± 0.5	2.8 ± 0.5	Control Mean ± SD	
0.0003	1.5 ± 0.3	2.6 ± 0.3	2.5 ± 0.6	Simvastatin Mean ± SD	IL-1β
0.0169	1.7 ± 0.4	2.5 ± 0.3	2.4 ± 0.6	Atorvastatin Mean ± SD	
0.0003	1.7 ± 0.2	2.5 ± 0.3	2.5 ± 0.8	Rosuvastatin Mean ± SD	
0.0004	3.2 ± 0.1	2.4 ± 0.4	2.2 ± 0.3	Chitosan-treated Mean ± SD	
0.0004	3.1 ± 0.6	2.5 ± 0.5	2.1 ± 0.3	Control Mean ± SD	

## Data Availability

The data presented in this study are available upon request from the corresponding authors. The data are not publicly available due to institutional policy and ethical restrictions.

## Abbreviations

The following abbreviations are used in this manuscript:

IL	Interleukin
IL-1β	Interleukin-1 beta
IL-6	Interleukin-6
IL-1 α	Interleukin-1 alpha
VEGF	Vascular endothelial growth factor
t-PA	Tissue plasminogen activator
PAI-1	Plasminogen activator inhibitor-1
PBS	Phosphate-buffered saline
ELISA	Enzyme-linked immunosorbent assay
TNF-α	Tumor necrosis factor-alpha
MMP-9	Matrix metalloproteinase-9
SPSS	Statistical Package for the Social Sciences
SD	Standard deviation
